# Herbal Bioenhancers in Veterinary Phytomedicine

**DOI:** 10.3389/fvets.2018.00249

**Published:** 2018-10-10

**Authors:** Begum Yurdakok-Dikmen, Yagmur Turgut, Ayhan Filazi

**Affiliations:** Department of Pharmacology and Toxicology, Faculty of Veterinary Medicine, Ankara University, Ankara, Turkey

**Keywords:** herbal bioenhancers, veterinary medicine, bioenhancing mechanisms, herb-drug interactions, phytomedicine

## Abstract

Herbal bioenhancers are active phytomolecules that increase the bioavailability, bioefficacy and biological activity of various drugs when coadministered at low concentrations. These valuable compounds reduce the dose, increase the treatment rate, decrease the treatment duration, drug resistance or related adverse reactions which have economical implications in livestock and pet medicine. Eventhough the concept of herbal bioenhancers are known for years through Ayurvedic medicine, the underlying mechanisms remains unclear. The main mechanisms involved are related to drug absorption (effect on solubility, drug efflux and transport proteins, increased permeability in gastrointestinal system) and drug metabolism (inhibition/induction of drug metabolysing enzymes, thermogenic effect). Due to species specific differences in these mechanisms, corresponding data on human and laboratory animal could not be attributed. As multidrug resistance is a major treat to both human and animal health, within “One Health” concept, efficient therapeutical strategies are encouraged by authorities, where focus on herbal supplements as a vast unexploited field remains to be researched within “Bioenhancement Concept.” This review brings insight to mechanims involved in bioenhancing effect, examples of herbal extracts and phytoactive compounds and their potential in the veterinary medicine including different classes of drugs such as antibiotics, anticancerous, antiviral, and antituberculosis.

## Introduction

Herbal bioenhancers (biopotentiers) are facilitating phytomolecules, which are able to promote and augment the biological activity including the bioavailability and bioefficacy of a particular drug or nutrient at low doses; where no typical pharmacological activity of its own at the dose used is present (1).

From the available literature, the concept of herbal bioenhancers is mainly attributed to Ayurvedic medicine (2). In Ayurveda, the term “Yogvahi” is referred as herbs that are able to increase or potentiate the plasma concentration of drug. Piperine from black pepper is the first example of Yogvahi which is scientifically validated. As in form of Trikatu (an Ayurvedic preparation) a mixture of black pepper, long pepper and ginger is prescribed as an ailment for various diseases (3).

As herbal drugs are mainly administered through oral routes, biodegredation in stomach or gut lumen occurs where membrane permeability and metabolism are the key contributors. This eventually lowers the free drug/pharmacologically active compound concentration in the systemic circulation and the efficacy is decreased. In order to increase the bioavailability rate, change in molecular structure and constituents of dosage form effect are studied extensively especially for oral route administered drugs (4). For this, formulation scientists adopted various strategies to enhance drug absorption; such as micronization, nanocrystals (ball-mining, dense gas), solid solutions, self dispersing solid solutions with surfactants, nanoparticles, lipid solutions, self-emulsifying drug delivery systems, surfactant-cosolvent systems (5). Meanwhile, these formulation systems are mainly designed for isolated active compounds as formulations and not for whole plant extracts. Therefore, herbal bioenhancers with different mechanism of action are favored for increasing the bioavailability of herbal preparations. Besides increase in bioavailability, bioenhancers act receptor for drug molecules, potentiate conformational interaction and make the target cells more receptive to drugs (6).

## Mechanism of action

The major mechanisms involved in bioenhancing property of plant extracts and phytomolecules are summarized as follows:

1. Alteration in the activity of enzymatic systems such as suppression of cytochrome P450 enzymes and its izoenzymes (piperine, naringin, gallic acid, quercetin) or stimulation of gamma glutamyl transpeptidase (GGT) activity (resulting uptake of aminoaids)
2. Alteration of drug transporters such as inhibition of P-glycoprotein efflux pump (caraway, sinomenine, genistein)
3. Cholagogues effect (promotion of bile into intestine) such as liquorice
4. Thermogenic/Bioenergetic effects leading increased metabolism rate and increased gastric motility such as garlic, ginger, turmeric
5. Regulation of gastrointestinal tract through reduction of hydrochloric acid secretion (Aloes, niaziridin, ginger, liquorice), inhibition of gastric emptying time, gastrointestinal transit and intestinal motility (Alliums, tea, liquorice), increase in gastrointestinal blood supply, modification in gastrointestinal tract epithelial cell membrane permeability.
6. Change physicochemical properties (hydrophobicity, pKa, solubility) such as phytosome formulations
7. Effect target drug receptors

### Bioenhancing through enzymatic alterations

Herbal medicines are combination of biologicaly active compounds; where metabolism of these several compounds might occur with the similar mechanism of the administered drug; leading interaction and eventually inhibition/increase of free drug metabolizing enzymes or transporters. The change in the expression of these proteins, or physical/chemical/pharmacological competition, eventually effects the free drug/metabolite concentration and the pharmakokinetic parameters leading altered pharmacological effects. Several herbs were found to interact cytochrome p450, the major microsomal enzyme for drug metabolism/detoxification, which has high polymorphisms in both human and companion animals (7). As CYP and UDPs are the core for phase I metabolism of many drugs, nutrients, endogenous substances, and environmental toxins; alteration of the expression or the functionality of these enzymes plays a crutial role in the efficacy of the therapy or the progress of the toxicity (8). Inhibition will lead fewer drug molecules to be metabolyzed with an increased concentration of untransformed drug passing from gut into the blood. Major isoenzymes among CYPs, involved in the metabolism of drugs in humans are CYP3A4, 2D6 and 2C9 family (9). Eventhough orthologue families are present in different animal species with structural similarities and substrate ranges comparable to humans; the species specific differences should be well considered for bioenhancing through enzymatic alterations. For instance, the orthologue enzyme to CYP3A4 in humans, is CYP3A12 in dogs and for CYP2D6 is CYP2D15. Also, polymorphism/genotypic variations (leading deficiencies or fold expressions) are common in different dog breeds for CYP1A, CYP2B11, CYP2C, CYP2D15 families (10). Therefore the clinically proven bioenhancing phytomolecules in human might not resemble veterinary medicine.

First purified bioenhancer molecule piperine were found to inhibit different forms of cytochrome p450 (CYP) (especially CYP3A4) (11) and hepatic/intestinal UDP-glucuronosyltransferases (UDP); while this inhibition is dependent on the administration route, dose and duration of exposure (12). Various studies reveal the potentiating effect of piperine in drug bioavailability and bioefficacy for laboratory animals such as rats, mice and rabbit along with human volunteers (11); while few are available for veterinary medicine. Oral administration of Trikatu, an Ayurvedic blend of equal parts of the fruits of Black Pepper (*Piper nigrum*), Long Pepper (*Piper longum*), and the rhizomes of Ginger (*Zingiber officinale*), increased the plasma concentration and the bioavailability of pefloxacin (fluoroquinolone antibiotic) in Gaddi goats (13). Similar increase in bioavailability and alterations of pharmacokinetics was observed for oxytetracycline treated hens which received oral *Piper longum* administration seven days prior to treatment (14).

St. John's wort (SJW-*Hypericum perforatum*), promising anxiolytic in pets were found to induce CYP3A4, CYP2C19, CYP2C9, CYP1A2, CYP2B6, CYP2E1; where decreased plasma levels of the substrates of many common drugs including antihistamines, antivirals, CNS drugs, immunosuppresants, statins, and chemotherapeutics are present with co or pretreatments such as increased warfarin and nifedipine clearance (15). The repeated administration of SJW were found to affect the pharmacokinetic profile with decreased maximum whole-blood concentration and area under the curve (AUC) in dogs treated by cyclosporine (16).

Homeopathic tinctures of *Ginkgo biloba* is used in all food producing species for improving the growth, slaughter performance and immune index; and pets for various ailments including neurological conditions and hormonal imbalances (17). *G. biloba* were found to induce various hepatic CYP enzymes, especially CYP2B-type enzymes in rats (18). *G. biloba* exerting a significant inductive effect on CYP2C19, were found to induce omeprazole hydroxylation in a CYP2C19 genotype-dependent manner decreasing its potential efficacy in human (19). G. biloba increased the AUC of raltegravir, an HIV integrase inhibitor in humans by 21% and Cmax by 44% which is favorable (20). Meanwhile, these examples with reduced AUCs in humans are mainly at doses higher than the recommended standardized extracts by European Pharmacopeia (21). In an *in vitro* microsomal study using male Beagle dogs, *G. biloba* extract inhibited CYP2C8, CYP2C9, and CYP2C19 enzyme, while no significant effect was present with coadministration of cilostazol where CYP2C is responsible for its metabolism (22).

The increase of bioavailability of several drugs including calcium channel blockers, benzodiazepines, and statins by grapefruit juice is mainly attributed to its furanocoumarin content which irreversibly inhibits CYP3A; where normal consumption inhibits only in the enterocyte cells lining the small intestine; while hepatic CYP remain unaffected unless at high concentrations (23). Meanwhile grapefruit juice has still potential on some orally-administered drugs that are metabolized by CYP3A and normally undergo extensive presystemic extraction like cyclosporine to increase the efficacy and reduce the costs. *In vitro* study using Beagle dog liver microsomes showed that grapefruit juice, lyophilized grapefruit juice, and powdered whole grapefruit inhibit cytochrome P450-mediated triazolam hydroxylation which is used as a surrogate for cyclosporine oxidation (23).

### Bioenhancing using phytosome formulations

Among herbal drug formulations, phytosomes as facilitating bioenhancing phytomolecules were recently introduced. Phytosomes, form a complex between a natural product and natural phospholipids; where the plant component and phosphatidylcholine form 1:1 or 1:2 molecular complex involving hydrogen bonds. The dual solubility property of this complex results in an increase in absorption and bioavailability compared to liposomes. Standardized plant extracts or polar active compounds (flavonoids, terpenoids, tannins, xanthones) in phytosome has improved lipid solubility. They also protect the active ingredients from destruction in stomach by gastric juice and gut bacteria leading better bioavailability (24, 25). Therefore, overall phytosomes improve absorption, increase biological activity and delivery to target tissue (26).

*Silybum marianum* (milk thistle) contains beneficial flavonoids (silybin, silydianin and silchristin) used for hepatoprotective effects, but it is poorly absorbed through oral routes. Meanwhile; phytosomal silybin (silybin-phospholipid complex) is more rapidly absorbed, lowering the conventional dose of slymarin; which improves the biological effects with reduced adverse/toxic effects. This compound is also used in cancer prevention and treatment (prostate cancer) and management of chronic iron overload (27, 28). Another example is centenella (*Centella asiatica* L.); where the triterpenes form complex with soyphospholipids a phytosome form which increases the oral bioavailability and decrease interaction with bile salts. Centenella phytosomes are used especially in cosmetics for its properties such as stimulation of collagen biosynthesis and modulate the metabolism in connective tissue along with reduction of increased capillary permeability. Centenella phytosomes were also found to induce antiinflammatory effect through NF-κB signaling inhibition in a mouse model of phthalic anhydride-induced atopic dermatitis; confirming its potential use in atopic dermatitis treatment (29).

### Bioenhancing through transporter protein alterations

Among transporter proteins P-glycoprotein (P-gp) acts as a physiological barrier found in the apical surface of epithelial cells adrenal gland, endothelial cells of the blood brain barrier (BBB) and in the surface of many neoplastic cells where it contributes of many drugs from the blood into the intestinal lumen. The inhibition of this efflux pump as an attractive therapeutic strategy through interference of the protein binding site, ATP hydrolysis or alteration of cell membrane lipid integrity, improves the delivery of therapeutic agents and increase the intracellular concentrations of drugs (30). Due to high prevalance of side effects of commercially available synthetic P-gp inhibitors, plant based alternatives are seeked for future drug candidates. Some active compounds of plants were shown to have inhibitory effect confirmed by *in vitro* and *in vivo* studies. Among these, flavonoids (3,7-dihydroxyflavone; 2′,4′-dihydroxychalcone from *Zuccagnia punctata*, quercetin from *Ginkgo biloba*; rutin from *Ruta graveolens*; genistein from vegetables; kaempfero from *Kaempferia galanga* L. root; icaritin from *Herba epimediu*; baicalein from the roots of *Scutellaria*; biochanin A from the bark of *Aesculus hippocastanum* L., silymarin from the seeds of milk thistle, wogonin from the roots of *Scutellaria baicalensi* Georgi.), alkaloids (glaucine from the stems of *Corydalis yanhusuo*, cepharanthine from the roots of *Stephania cepharantha*, pyrrolidine from *Piper boehmeriifolium*, tetrandrine and fangchinoline from the root of *Stephania tetranda*, indole-3-carbinol from Brassica, pervilleines B and C from *Erythroxylum pervillei*, stemocurtisine and oxystemokerrine from the roots of *Stemona aphylla* and *Stemona burkillii*), coumarins (cnidiadin from *Tordylium apulum*, clausarin from *Citrus sinensis*, galbanic acid from the roots of *Ferula szowitsiana*, farnesiferol A from the roots of *Ferula persica*, GUT-70 from stem bark of *Calophyllum brasiliense*), terpenoids ((R)-(+)-citronellal essential oil from *Zanthoxyli fructus*, abietic acid from pine and conifers, Glycyrrhetic acid from *Glycyrrhizae radix*, euphomelliferine and euphomelliferenes A from *Euphorbia mellifera*, Euphorbia factor L10 from *Euphorbia lathyris*, euphoportlandols A and B from *Euphorbia portlandica*) and steroids (Paris saponin VII from *Trillium tschonoskii*, ginsenoside Rg_3_ and protopanaxatriol ginsenosides from *Panax ginseng*, gracillin and polyphyllin D from *Paris polyphylla*) were promising (31).

Meanwhile, genetically mediated P-gp deficiencies in dogs (several breeds including Longhaired whippet, Collie, Australian Shephard, Silken Woundhound are heterozygous for ABCB1-1Δ) contribute increased brain penetration of P-gp substrates such as macrocyclic lactones, loperamide, acepromazine, butorphanol, vincristine leading neurotoxicity (32). The relation between the P-gp inhibitors and genetic deficiencies should be as well considered. Such as, the potent inhibition of the drug transporter P-gp by the oral flea preventative spinosad was shown in canine lymphoid cell-line GL-1 and the P-gp overexpressing subline GL-40, where kinetics of ivermectin, cyclosporin, verapamil, loperamide and ketoconazole were altered (33). As currently available P-gp inhibitors can not discriminate between the expression of this protein in normal tissues and cancerous tissues, intrinsic toxicity (cytotoxicty) of the drugs are common which would eventually harm the patient. Therefore, monitoring systems are required for precautionary measures of such severe interactions (32). Contrary to dogs, few studies are available for the functional modeling of feline P-gp (use of feline lymphoma cells) for further plant-drug interactions (34).

### Bioenhancing through cholagogue/choleretic effect

Cholagogues stimulate the release and secretion of bile from gallbladder, aiding in the digestion and absorption of lipids along with absorption from drugs from the gastrointestinal tract. Mainly, choleretics improve bile flow and cholagogues stimulate gallbladder motility. They are mainly used in cholecystitis and cholelithiasis diseases along with cases where spasmolytic action is required in intestines. Drugs with low water solubility are well solubilized in bile salt-phospholipid micelles; where they are transported to the intestinal wall with increased bioavailability. Bile acids also serve as signaling molecules through binding the nuclear receptors for the control of hepatocyte metabolism; which would also have an effect on bioavailability (35). Medicinal plants with cholagogic/choleretic properties include chamomile (*Chamomilla recutita*), elecampain (*Inula helenium*), dandelion (*Taraxacum officinalis*), St. John's wort (*Hypericum perforatum*), Artemisia sp, yarrow (*Achillea millefolium*), rosemary (*Rosmarinus officinalis*), chelidonium (*Chelidonium majus*). Essential oils of liquorice, coriander, turmeric, black pepper, red chili, cumin, onion, peppermint also have choleretic properties.

### Bioenhancing through thermogenesis

Thermogenic agents increase the utilization of ATP or uncoupling oxidative phosphorylation of the reduced coenzymes. These compounds might mimic or antagonize hormones (activation of adrenergic, thyroid hormone or growth hormone receptors and the inhibition of glucocorticoid receptors), target miscellaneous intracellular mechanisms (modulation of transcriptional factors/enzymes to modulate mitochondrial biogenesis to promote fatty acid oxidation 36). Besides its major role in weight loss, increased cellular energy levels utilize the nutrients through varios mechanisms in digestion and gastrointestinal absorption. Natural compounds with thermogenic effects include berberine (AMPK activation), butein (Prdm4 induction), capsaicin (TrpV1 activation), 7,8-dihydroxyflavone (muscular TrkB activation), fucoxanthin (37).

## Selected herbal bioenhancers

### Long pepper (*piper longum*) and black pepper (*piper nigrum*)

Piperine, the major plant alkaloid present in *P. nigrum* Linn. (Black pepper) and *P. longum* Linn. (Long pepper), induce bioenhancing effects through inhibiton of P-gp, drug metabolyzing enzymes (arylhydrocarbon hydroxylase, uridine diphosphate, glucuronyl transferase, ethylmorphine-N-demethylase, 7-Ethoxycoumarin-O-deethylase, 3-Hydroxy-benzo(a)pyrene glucuronidation, UDP-glucose dehydrogenase, 5-lipoxygenase, cyclooxygenase-1, cytochrome P450), increased blood supply to gastrointestinal system tract, decreased hydrochloric acid secretion and other proteins/enzymes involved in their transport leading increased bioavailability ranging 30–200%. Pyrazinamide, phytoin, propranolol, theophylin, curcumin, rifampicin, amoxycillin, oxytetracycline, ciprofloxacin, and nevirapine are some examples that are bioenhanced by piperine and used in veterinary medicine (1, 38). For more, piperine decreases the cytotoxicity and genotoxicity of aflatoxin B1, through inhibition of metabolization pathways shown *in vitro* (39). In broiler chickens intoxicated by AFB1 (ingestion of 0.5 mg AFB1 kg^−1^ bw); piperine (60 mg kg^−1^) was able to reduce or even prevent the genotoxic and cytotoxic effects, presenting a safe option for the protection and supportive treatment of aflatoxicosis (40).

### Turmeric (*curcuma longa*)

Curcumin, the natural phenolic coloring component of *Curcuma longa* suppresses drug metabolyzing enzymes and P-gp to exert its bioenhancing properties; while it also influences multiple signaling pathways and antioxidant mechanisms for its other pharmacological effects (41). Curcumin were found to modulate (increase) the pharmacokinetics of drugs that are P-gp subtrates such as oral administration of celiprolol, midazolam and paclitaxel in rats (42, 43), marbofloxacin in broiler chickens (44).

### Ginger (*zingiber officinale*)

The rhizome extract of *Zingiber officinale* contain gingerols to be further converted into shogaols, zingerone and paradol; which regulates the intestinal functions to facilitate absorption increasing the absorption of drugs along and have cholagogeus effects (45). It inhibits the human CYP2C9, CYP2C19, CYP2D6, and CYP3A4 metabolic related reactions (46, 47). As mentioned previously, in Ayurvedic medicine “Yogavahi,” the basis for modern bioenhancement theories, include “trikatu” preparation containing the mixture of *Piper longum* (long pepper), *Piper nigrum* (black pepper), and *Zingiber officinale* (ginger) (48). Through piperine combination, an increase of bioavailability is evident (49). Ginger significantly influenced the pharmakokinetic profile of pefloxacin when coadministered in rabbits, with an increase in maximal concentration, AUC and halflife (50). It was also shown to increase the bioavailability of methotrexate, 5-fluorouracil and acyclovir (49, 51).

### Cumin (*cuminum cyminum*)/caraway (*carum carvi*)

Novel flavonoid glycoside isolated from cumin (3′,5-dihydroxyflavone 7-O-β-d-galacturonide-4′-O-β-d-glucopyranoside) were shown to enhance the peak concentration and AUC of rifampicin at coadministration (52). Caraway seed was also found to enhance the plasma levels of antitubercular drugs: rifampicin (RIF), pyrazinamide (PZA), and isoniazid (INH), when co-dosed in combination in Wistar rats (53). Bioenhancing effect of cumin/caraway was related to the permeation-enhancing property across the small intestinal absorptive surface. *Carum carvi* extracts were also found to inhibit the 2, 3, 7, 8-tetrachloro-dibenzo-p-dioxin-dependent gene expression of cytochrome P450 1A1 in the rat hepatoma cells (54), therefore it may affect the drugs metabolyzed by CYP1A1 such as chlorzoxazone, theophylline, bufuralol.

### Black cumin (*nigella sativa*)

The methanolic extracts of *Nigella sativa* Linn. (Ranunculaceae), indigenous in Mediterranean region, was found to improve the intestinal permeability of amoxicillin in *in-vitro* experiments using excised rat intestinal segments in a dose-dependent manner (55).

Besides improving the intestinal permeability, the fatty acids of black cumin (especially eicosadeinoic acid) were found to inhibit the P-gp activity *in silico* against the primary amino acid sequence of P-gp from rats leading increased bioavailability of drugs (56). Contrary to *in silico* analysis, black cumin is associated with the activation of intestinal P-gp and/or CYP3A4, due to its ability to decrease the bioavailability of cyclosporine (cyclic polypeptide used as immunosuppressant in organ transplantation) in coadministration (57). Black cumin induced no significant effect on the theophylline pharmakokinetics in Beagle dogs indicating its lack of affinity to CYP1A2 activity; meanwhile fenugreek and garden cress decreased the bioavailability (58).

### Morning glory plant (*ipomoea* spp.)

Lysergol is an alkaloid of the ergoline family isolated from the morning glory family and other higher plants. It was found to increase the bioavailability of curcumin. In order to evaluate the mechanism of this enhancing bioavailability related to curcumin, its effect on human P-gp and Breast Cancer Resistance Protein (BCRP) as major efflux transporters, were evaluated using *in situ* permeation and *in vitro* pharmakokinetic studies using specific substrates (digoxin for P-gp probe, sulfasalazine for BCRP probe) and inhibitors (verapamil for P-gp and pantoprazole for BCRP). The results indicate that the bioavailability enhancing potential of lysergol was attributed to BCRP efflux transport system (59). Lysergol also enhanced the oral bioavailability of berberine in rats; while mechanism of action of lysergol was not confirmed eventhough it was related to P-gp inhibition (60).

### Garlic (*alllium sativum*)

Garlic (*Alllium sativum*) has been used thousands of years mainly for the treatment of hypercholesterolaemia and prevention of arteriosclerosis (61). Allicin is an important allyl sulfur metabolite isolated from garlic and enhances the fungicidal activity of Amphotericin B against pathogenic fungi such as *Candida albicans, Aspergillus fumigatus* and yeast *Saccharomyces cerevisiae* (62–65) and antibacterial activity of β-lactams (cefazolin, oxicillin, and cefaperazone) tested at subinhibitory concentrations against *Staphylococcus* spp. and *Pseudomonas aeruginosa* (66). Also, garlic oils exhibited synergistic antifungal effects when combined with ketoconazole *in vitro* (67). It has been reported that garlic extracts inhibit some CYP enzymes (CYP2C9, CYP2C19, CYP3A4, and CYP3A5) *in vitro* but do not affect P-gp (68). Conversely, in a clinical study was demonstrated that garlic powder has no effect on CYP3A4 (69). Moreover, garlic oil can selectively inhibit CYP2E1 (70). In a later *in vivo* study, garlic extract was demonstrated to have stimulatory effects on both efflux and uptake transporters (71). These effects increase the risk of interactions with some drugs having narrow therapeutic index. For example, ajoene isolated from garlic is a weak inhibitor of platelet aggregation in baboons (72) and rats (73) when combined with antiplatelet dipyridamole. Careful addition of garlic homogenate in moderate doses (250 mg kg^−1^) in propranolol treatment might result in beneficial effect during treatment of hypertension in animals with myocardial damage. Garlic induces a reduction in systolic blood pressure, cholesterol, triglycerides and glucose levels and an increase in the bioavailability and half-life along with decrease in the clearance and elimination rate constant of propranolol when ingested. Garlic also improved the survival and cardiac function in captopril or hydrochlorothiazide-treated rats with myocardial infarction (74–76). Overall, the bioenhancing effects of garlic are mainly attributed to its inhibitory effect of some CYP enzymes along with physiological changes in circulation leading a decrease inclearance and elimination of drugs.

### Liquorice (*glycyrrhiza glabra*)

In traditional medicine, *Glycyrrhiza glabra* or liquorice are used to treat peptic ulcer and cough (77). Glycyrrhizin is an active substance derivated from liquorice and enhances the transport across the intestinal membrane of some antimicrobials (rifampicin, tetracycline, nalidixic acid, and ampicillin), vitamin B1, and vitamin B12 (6). Moreover, glycyrrhizin increases the Taxol effect by 5 times, inhibiting the division of the cell (MCF-7 cancer cell line) (78). Glycyrrhizin did not induce an effect on CYP3A4; meanwhile nine other compounds isolated from the extract of licorice including (3R)-vestitol, 4-hydroxyguaiacol apioglucoside and liquiritigenin 7,4′-diglucoside showed potent CYP3A4 activity (79). Another metabolite of liquorice, glycyrrhizic acid, does not inhibit the intestinal efflux transporter P-gp *in vitro* (80). However, a study suggested activator effects for both glycyrrhizin and glycyrrhetinic acid on P-gp function *in vitro* (81). In a study reported that a metabolite of liquorice, glabridin, has inhibitory effects on CYP3A4, 2B6, and 2C9 (82). Other metabolite of liquorice, glycyrrhetinic acid exhibits inhibitory effects on CYP3A, 2C9, and 2C19 *in vitro* and *in vivo* (83) and induces UDP glucuronosyltransferases in rat liver *in vivo* (84). Glycyrrhizin and glycyrrhetinic acid induced inhibitory effect on nucleoside transporters, thereby reduced bioavailability of ribavirin due to reduction in ribavirin transportation in intestinal tract (85).

### Aloe (*aloe vera*)

Aloe, a perennial and succulent xerophyte is widely been used in both human and veterinary medicine for its immunomodulatory, wound and burn healing, hypoglycemic, anticancer, gastro-protective, antifungal, and anti-inflammatory effects (86). The ethanolic extract of Aloe vera were found to augment the hypoglycemic effect of glipizide in streptozotocin induced diabetic rats (87). Due to its cytoprotective effects on gastric mucosa through induction of endogenous prostaglandin production, concominant use of *Aloe vera* and pantoprazole for the gastroesophageal reflux symptoms in mustard gas victims were found to be improved compared to single treatments (88). Meanwhile, aloe ingestion were found to activate the functions of P-gp and CYP3A, decreasing the cyclosporine bioavailability in a rat model (89); therefore a decrease in the bioavailability of the related absorbed/metabolyzed drugs could be expected.

## Conclusion

Medicinal plants with bioenhancing potentials are not yet extensively explored especially in veterinary medicine. Bioenhancers are valuable tools to reduce the dose of the drugs and the duration of the treatment which is especially important for economic outcomes in livestock medicine along with their beneficial ecological implications. These compounds also reduce the drug resistance or related adverse reactions and toxicity which is especially important in anticancer drugs. Meanwhile drug-herb interactions are complex where species specific differences along with case specific differences play an important role. They are expected to produce least (or even no) pharmacological effects at the treatment concentrations; while they would increase the bioavailability and the efficacy of the target drug or nutraceutical (1, 6). Eventhough the major known example among herbal bioenhancers is piperine, various phytoactive compounds (capsaicin, quercetin, naringin) and plant extracts are yet to be exploited with their mechanism of action and complex drug interactions.

## Author contributions

All authors listed have made a substantial, direct and intellectual contribution to the work, and approved it for publication.

### Conflict of interest statement

The authors declare that the research was conducted in the absence of any commercial or financial relationships that could be construed as a potential conflict of interest.

## References

[1] Dudhatra GB Mody SK Awale MM Patel HB Modi CM Kumar A. A comprehensive review on pharmacotherapeutics of herbal bioenhancers. Sci World J. (2012) 2012:637953. 10.1100/2012/637953 23028251 PMC3458266

[2] Singh R Devi S Patel JH Patel UD Bhavsar SK Thaker AM Indian herbal bioenhancers: a review. Pharmacog Rev. (2009) 3:90–91.

[3] Jhanwar B Gupta S Biopotentiation using herbs: Novel technique for poor bioavailable drugs. Int J PharmTech Res. (2014) 6:443–54.

[4] Musther H Olivares-Morales A Hatley OJD Liu B Rostami HA. Animal versus human oral drug bioavailability: do they correlate? Eur J Pharm Sci. (2014) 57:280–91. 10.1016/j.ejps.2013.08.018 23988844 PMC4107270

[5] Gupta S Kesarla R Omri A. Formulation strategies to improve the bioavailability of poorly absorbed drugs with special emphasis on self-emulsifying systems. ISRN Pharm. (2013) 2013:848043. 10.1155/2013/848043 24459591 PMC3888743

[6] Jain G Patil UK Strategies for enhancement of bioavailability of medicinal agents with natural products. Int J Pharm Sci Res. (2015) 6:5315–24.

[7] Liu MZ Zhang YL Zeng MZ He FZ Luo ZY Luo JQ. Pharmacogenomics and herb-drug interactions: merge of future and tradition. Evid Based Compl Alt Med. (2015) 2015:1–8. 10.1155/2015/321091 25821484 PMC4363646

[8] Teo YL Ho HK Chan A. Metabolism-related pharmacokinetic drug-drug interactions with tyrosine kinase inhibitors: current understanding, challenges and recommendations. Br J Clin Pharm. (2015) 79:241–53. 10.1111/bcp.12496 25125025 PMC4309630

[9] Kumar S Sharma R Roychowdhury A. Modulation of cytochrome-P450 inhibition (CYP) in drug discovery: a medicinal chemistry perspective. Cur Med Chem. (2012) 19:3605–21. 10.2174/092986712801323180 22680629

[10] Fleischer S Sharkey M Mealey K Ostrander EA Martinez M. Pharmacogenetic and metabolic differences between dog breeds: their impact on canine medicine and the use of the dog as a preclinical animal model. AAPS J. (2008) 10:110–9. 10.1208/s12248-008-9011-1 18446511 PMC2747081

[11] Mhaske DB Sreedharan S Mahadik KR Role of piperine as an effective bioenhancer in drug absorption. Pharm Analyt Acta (2018) 9:1–4. 10.4172/2153-2435.1000591

[12] Lee SH Kim HY Back SY Han H-K. Piperine-mediated drug interactions and formulation strategy for piperine: recent advances and future perspectives. Exp Opin Drug Met Toxicol. (2018) 14:43–57. 10.1080/17425255.2018.1418854 29250980

[13] Dama MS Varshneya C Dardi MS Katoch VC. Effect of trikatu pretreatment on the pharmacokinetics of pefloxacin administered orally in mountain Gaddi goats. J Vet Sci. (2008) 9:25–9. 10.4142/jvs.2008.9.1.25 18296885 PMC2839109

[14] Singh M Varshneya C Telang RS Srivastava AK. Alteration of pharmacokinetics of oxytetracycline following oral administration of *Piper longum* in hens. (2005) J Vet Sci. 6:197–200. 16131821

[15] Grimstein M Huang S-M. A regulatory science viewpoint on botanical–drug interactions. J Food Drug Anal. (2018) 26:S12–25. 10.1016/j.jfda.2018.01.013 29703380 PMC9326881

[16] Fukunaga K Orito K. Time-course effects of St John's wort on the pharmacokinetics of cyclosporine in dogs. J Vet Pharm Ther. (2012) 35:446–51. 10.1111/j.1365-2885.2011.01348.x 22091645

[17] Araujo JA Landsberg GM Milgram NW Miolo A. Improvement of short-term memory performance in aged beagles by a nutraceutical supplement containing phosphatidylserine, *Ginkgo biloba*, vitamin E, and pyridoxine. Canad Vet J. (2008) 49:379–85. 18481547 PMC2275342

[18] Umegaki K Saito K Kubota Y Sanada H Yamada K Shinozuka K. *Ginkgo biloba* extract markedly induces pentoxyresorufin O-dealkylase activity in rats. Jap J Pharm. (2002) 90:345–51. 12501011 10.1254/jjp.90.345

[19] Yin OQ Tomlinson B Waye MM Chow AH Chow MS. Pharmacogenetics and herb-drug interactions: experience with g*inkgo biloba* and omeprazole. Pharmacogenetics (2004) 14:841–50. 10.1097/00008571-200412000-00007 15608563

[20] Blonk M Colbers A Poirters A Schouwenberg B Burger D. Effect of *Ginkgo biloba* on the pharmacokinetics of raltegravir in healthy volunteers. Antimicrob Agents Chemother. (2012) 56:5070–5. 10.1128/AAC.00672-12 22802250 PMC3457394

[21] Unger M. Pharmacokinetic drug interactions involving g*inkgo biloba*. Drug Metab Rev. (2013) 45:353–85. 10.3109/03602532.2013.815200 23865865

[22] Chung H Kim N-S Kim E-J Kim T-K Ryu K-H Lee B-Y Negligible effect of *Ginkgo biloba* extract on the pharmacokinetics of cilostazol. Biomol Ther. (2009) 17:311–7. 10.4062/biomolther.2009.17.3.311

[23] Hanley MJ Cancalon P Widmer WW Greenblatt DJ. The effect of grapefruit juice on drug disposition. Expert Opin Drug Metab Toxicol. (2011) 7:267–86. 10.1517/17425255.2011.553189 21254874 PMC3071161

[24] Hetal T Bindesh P Sneha T A review on techniques for oral bioavailability enhancement of drugs. Int J Pharm Sci Rev Res. (2010) 4:203–23.

[25] Hogervorst CJ Atanacković M Bursać M Miljić U Polyphenols nutraceutical and functional food components. In: Galanakis CM, editor. Nutraceutical and Functional Food Components. Effects of Innovative Processing Techniques. Academic Press (2017). p. 203–258.

[26] Mukherjee PK Venkatesh M Gantait A Ayurveda in modern medicine: development and modification of bioactivity. In: Mander L, Liu HW, editors. Comprehensive Natural Product Chemistry-II. 1st edn. Elsevier (2010) p. 479–507.

[27] Bergman Å Heindel JJ Jobling S Kidd KA Zoeller RT State of the Science of Endocrine Disrupting Chemicals 2012 Summary for Decision-Makers. WHO (World Health Organization)/UNEP (United Nations Environment Programme). Geneva:UNEP/WHO (2013) Available online at: http://www.who.int/ceh/publications/endocrine/en/

[28] El-Gazayerly ON Makhlouf AIA Soelm AMA Mohmoud MA. Antioxidant and hepatoprotective effects of silymarin phytosomes compared to milk thistle extract in CCl4 induced hepatotoxicity in rats. J Microencapsul. (2014) 31:23–30. 10.3109/02652048.2013.805836 23808477

[29] Ju Ho P Jun Sung J Ki Cheon K Jin Tae H Anti-inflammatory effect of *Centella asiatica* phytosome in a mouse model of phthalic anhydride-induced atopic dermatitis. Phytomed (2018) 43:110–9. 10.1016/j.phymed.2018.04.013 29747743

[30] Amin ML. P-glycoprotein inhibition for optimal drug delivery. Drug Target Insights (2013) 7:27–34. 10.4137/DTI.S12519 24023511 PMC3762612

[31] Yu J Zhou P Asenso J Yang XD Wang C Wei W. Advances in plant-based inhibitors of p-glycoprotein. J Enzyme Inhib Med Chem. (2016) 31:867–81. 10.3109/14756366.2016.1149476 26932198

[32] Mealey KL Fidel J. P-glycoprotein mediated drug interactions in animals and humans with cancer. J Vet Intern Med. (2015) 29:1–6. 10.1111/jvim.12525 25619511 PMC4858061

[33] Schrickx JA. *Spinosad* is a potent inhibitor of canine p-glycoprotein. The Vet J. (2014) 200:195–6. 10.1016/j.tvjl.2014.01.012 24582422

[34] van Beusekom CD Lange R Schrickx JA A functional model for feline p-glycoprotein. J Vet Pharmacol Therap. (2015) 39:95–7. 10.1111/jvp.12248 26190674

[35] Holm R Müllertz A Mu H. Bile salts and their importance for drug absorption. Int J Pharm. (2013) 453:44–55. 10.1016/j.ijpharm.2013.04.003 23598075

[36] Clapham JC Arch JRS. Thermogenic and metabolic antiobesity drugs: rationale and opportunities. Diab Obes Metabol. (2007) 9:259–75. 10.1111/j.1463-1326.2006.00608.x 17391151

[37] Song NJ Chang SH Li DY Villanueva CJ Park KW. Induction of thermogenic adipocytes: molecular targets and thermogenic small molecules. Exp Mol Med. (2017) 49:e353. 10.1038/emm.2017.70 28684864 PMC5565954

[38] Kesarwani K Gupta R Mukerjee A. Bioavailability enhancers of herbal origin: an overview. Asian Pac J Trop Biomed. (2013) 3:253–66. 10.1016/S2221-1691(13)60060-X 23620848 PMC3634921

[39] Reen RK Wiebel FJ Singh J. *Piperine* inhibits aflatoxin B1-induced cytotoxicity and genotoxicity in V79 chinese hamster cells genetically engineered to express rat cytochrome P4502B1. J Ethnopharmacol. (1997) 58:165–73. 10.1016/S0378-8741(97)00104-9 9421252

[40] da Silva Cardoso V Vermelho AB Ribeiro de Lima CA Mendes de Oliveira J Freire de Lima ME Pinto da Silva LH Antigenotoxic effect of piperine in broiler chickens intoxicated with Aflatoxin B1. Toxins (2016) 8:E316 10.3390/toxins8110316 27809242 PMC5127113

[41] Hatcher H Planalp R Cho J Torti FM Torti SV. Curcumin: from ancient medicine to current clinical trials. Cell Mol Life Sci. (2008) 65:1631–52. 10.1007/s00018-008-7452-4 18324353 PMC4686230

[42] Sharma A Magotra A Nandi U Singh G Enhancement of paclitaxel oral bioavailability in swiss mice by four consecutive days of pre-treatment with c*urcumin*. Indian J Pharm Educ Res. (2017) 51:S566–70. 10.5530/ijper514s84

[43] Zhang W Tan TM Lim CLY. Impact of c*urcumin*-induced changes in p-glycoprotein and CYP3A expression on the pharmacokinetics of peroral celiprolol and midazolam in rats. Drug Metab Disp. (2007) 35:110–5. 10.1124/dmd.106.011072 17050652

[44] Abo-El-Sooud K Samar MM Fahmy M-AF Curcumin ameliorates the absolute and relative bioavailabilities of marbofloxacin after oral administrations in broiler chickens. Wulfenia (2017) 24:284–97.

[45] Dhanik J Arya N Nand V A review on *Zingiber officinale*. J Pharmacog Phytochem J. (2017) 174:174–84.

[46] Kim IS Kim SY Yoo HH. Effects of an aqueous-ethanolic extract of ginger on cytochrome P450 enzyme-mediated drug metabolism. Pharmazie (2012) 67:1007–9. 10.1691/ph20122595 23346764

[47] Li M Chen P Yue Q Li J Chu R Zhang W. Pungent ginger components modulates human cytochrome P450 enzymes *in vitro*. Acta Pharmacol Sin. (2013) 34:1237–42. 10.1038/aps.2013.49 23770984 PMC4003154

[48] Johri RK Zutshi U. An ayurvedic formulation ‘Trikatu' and its constituents. J Ethnopharm. (1992) 37:85–91. 10.1016/0378-8741(92)90067-2 1434692

[49] Ajazuddin AA Qureshi A Kumari L Vaishnav P Sharma M Saraf S. Role of herbal bioactives as a potential bioavailability enhancer for active pharmaceutical ingredients. Fitoter (2014) 97:1–14. 10.1016/j.fitote.2014.05.005 24862064

[50] So N Lz A Eo O Jm O The influence of ginger (*Zingiber officinale*) extract on the pharmacokinetic profile of perfloxacin. Int J Appl Res Nat Prod. (2013) 6:15–8.

[51] Qazi GN Bedi KL Johri RK Tikoo MK Tikoo AK (2002) WO2003049753A1. Available online at: https://patents.google.com/patent/WO2003049753A1/de

[52] Sachin BS Sharma SC Sethi S Tasduq SA Tikoo MK Tikoo AK. Herbal modulation of drug bioavailability: enhancement of rifampicin levels in plasma by herbal products and a flavonoid glycoside derived from *Cuminum cyminum*. Phytother Res. (2007) 21:157–63. 10.1002/ptr.2046 17128432

[53] Sachin B Monica P Sharma S Satti N Tikoo M Tikoo A. Pharmacokinetic interaction of some antitubercular drugs with caraway: implications in the enhancement of drug bioavailability. Hum Exp Toxicol. (2009) 28:175–84. 10.1177/0960327108097431 19734267

[54] Naderi-Kalali B Allameh A Rasaee MJ Bach HJ Behechti A Doods K. Suppressive effects of caraway (*Carum carvi*) extracts on 2, 3, 7, 8-tetrachloro-dibenzo-p-dioxin-dependent gene expression of cytochrome P450 1A1 in the rat H4IIE cells. Toxicol in vitro (2005) 19:373–7. 10.1016/j.tiv.2004.11.003 15713544

[55] Ali B Ali M Amin S Mir SR Enhancement of gut permeation of amoxicillin with *Nigella sativa* seed extract and its phytochemical screening. Chin J Nat Med. (2018) 6:125–30. 10.1016/S1875-5364(18)30038-4 29455727

[56] Ali B Jamal QMS Mir SR Shams S Al-Wabel NA Kamal MA. *In silico* analysis for predicting fatty acids of black cumin oil as inhibitors of p-glycoprotein. Pharmacog Mag. (2015) 11(Suppl 4):S606–10. 10.4103/0973-1296.172969 27013802 PMC4787096

[57] Al-Jenoobi FI Al-Suwayeh SA Muzaffar I Alam MA Al-Kharfy KM Korashy HM. Effects of *Nigella sativa* and *Lepidium sativum* on cyclosporine pharmacokinetics. BioMed Res Int. (2013) 2013:953520. 10.1155/2013/953520 23957013 PMC3730136

[58] Al-Jenoobi FI Ahad A Mahrous GM Al-Mohizea AM AlKharfy KM Al-Suwayeh SA. Effects of fenugreek, garden cress, and black seed on theophylline pharmacokinetics in beagle dogs. Pharm Biol. (2015) 53:296–300. 10.3109/13880209.2014.916312 25243874

[59] Shukla M Malik MY Jaiswal S Sharma A Tanpula DK Goyani R A mechanistic investigation of the bioavailability enhancing potential of *lysergol*, a novel bioenhancer, using *curcumin*. RSC Adv. (2016) 6:58933–42. 10.1039/C6RA09307H

[60] Patil S Dash RP Anandjiwala S Nivsarkar M. Simultaneous quantification of berberine and lysergol by HPLC-UV: evidence that lysergol enhances the oral bioavailability of berberine in rats. Biomed Chromat. (2012) 26:1170–5. 10.1002/bmc.2674 22213237

[61] Banerjee SK Maulik SK. Effect of garlic on cardiovascular disorders: a review. Nutr J. (2002) 1:4. 10.1186/1475-2891-1-4 12537594 PMC139960

[62] Jamindar D Patidar N Jain S Formulation and characterization of allicin-amphotericin-b liposomal gel for the treatment of fungal infections. J Drug Deliver Therap. (2017) 7:69–70. 10.22270/jddtv7i71590

[63] Mekala P Arivuchelvan A Bioenhancer for Animal Health and Production: A Review. Noto-Are Agriculture (2012) p. 1–6. Available online at: https://www.notoare.com/index.php/index/explorer/getPDF/11155755

[64] Ogita A Fujita KI Tanaka T. Enhancing effects on vacuole-targeting fungicidal activity of amphotericin B. Front Microbiol. (2012) 3:100. 10.3389/fmicb.2012.00100 22457662 PMC3307023

[65] Ogita A Fujita K Taniguchi M Tanaka T. Enhancement of the fungicidal activity of amphotericin B by a*llicin*, an allyl-sulfur compound from garlic, against the yeast *Saccharomyces cerevisiae* as a model system. Planta Med. (2006) 72:1247–50. 10.1055/s-2006-947203 16902870

[66] Cai Y Wang R Pei F Liang B-B. Antibacterial activity of allicin alone and in combination with β-Lactams against *Staphylococcus* spp and *Pseudomonas aeruginosa*. J Antib. (2007) 60:335–8. 10.1038/ja.2007.45 17551215

[67] Pyun MS Shin S. Antifungal effects of the volatile oils from *allium* plants against *Trichophyton* species and synergism of the oils with ketoconazole. Phytomed (2006) 13:394–400. 10.1016/j.phymed.2005.03.011 16716908

[68] Foster BC Foster MS Vandenhoek S Krantis A Budzinski JW Choudri S. An *in vitro* evaluation of human cytochrome P450 3A4 and P-glycoprotein inhibition by garlic. J Pharm Pharm Sci. (2001) 4:176–84. 11466175

[69] Cox MC Low J Lee J Walshe J Denduluri N Berman A. Influence of garlic (*Allium sativum*) on the pharmacokinetics of docetaxel. Clin Cancer Res. (2006) 12:4636–40. 10.1158/1078-0432.CCR-06-0388 16899612

[70] Izzo AA Ernst E. Interactions between herbal medicines and prescribed drugs. Drugs (2009) 69:1777–98. 10.2165/11317010-000000000-00000 19719333

[71] Berginc K Kristl A. The mechanisms responsible for garlic-drug interactions and their *in vivo* relevance. Curr Drug Metabol. (2013) 14:90–101. 10.2174/138920013804545188 21838705

[72] Teranishi K Apitz-Castro R Robson SC Romano E Cooper DKC. Inhibition of baboon platelet aggregation *in vitro* and *in vivo* by the garlic derivative, *ajoene*. Xenotransplant (2003) 10:374–79. 10.1034/j.1399-3089.2003.02068.x 12795687

[73] Wang Y Zou M Zhao N Ren J Zhou H Cheng G. Effect of diallyl trisulfide on the pharmacokinetics of dipyridamole in rats. Arch Pharmacol Res. (2011) 34:1957–64. 10.1007/s12272-011-1116-x 22139695

[74] Asdaq SM Inamdar MN. Pharmacodynamic interaction of garlic with hydrochlorothiazide in rats. Indian J Physiol Pharmacol. (2009) 53:127–36. 20112816

[75] Asdaq SM Inamdar MN. Pharmacodynamic interaction of captopril with garlic in isoproterenol-induced myocardial damage in rat. Phytother Res. (2010) 24:720–5. 10.1002/ptr3009 19830688

[76] Asdaq SM Inamdar MN. Pharmacodynamic and pharmacokinetic interactions of propranolol with garlic (*Allium sativum*) in rats. Evid Based Complement Alternat Med. (2011) 2011:824042. 10.1093/ecam/neq076 21792365 PMC3137651

[77] Menegazzi M Dipaola R Mazzon E Genovese T Crisafulli C Dalbosco M. *Glycyrrhizin* attenuates the development of carrageenan-induced lung injury in mice. Pharm Res. (2008) 58:22–31. 10.1016/j.phrs.2008.05.012 18590825

[78] Nazari S Rameshrad M Hosseinzadeh H. Toxicological effects of *glycyrrhiza glabra* (Licorice): a review. Phyto Res. (2017) 31:1635–50. 10.1002/ptr.5893 28833680

[79] Tsukamoto S Aburatani M Yoshida T Yamashita Y El-Beih AA Ohta T. CYP3A4 inhibitors isolated from *licorice*. Bio Pharm Bull. (2005) 28:2000–2. 10.1248/bpb.28.2000 16204965

[80] Nabekura T Yamaki T Ueno K Kitagawa S. Inhibition of p-glycoprotein and multidrug resistance protein 1 by dietary phytochemicals. Cancer Chemother Pharmacol. (2008) 62:867–73. 10.1007/s00280-007-0676-4 18204840

[81] Hou Y-C Lin S-P Chao P-DL. Liquorice reduced cyclosporine bioavailability by activating P-glycoprotein and CYP 3A. Food Chem. (2012) 135:2307–12. 10.1016/j.foodchem.2012.07.061 22980806

[82] Kent UM Aviram M Rosenblat M Hollenberg PF. The licorice root derived isoflavan glabridin inhibits the activities of human cytochrome P450S 3A4, 2B6, and 2C9. Drug Metabol Dispos. (2002) 30:709–15. 10.1124/dmd.30.6.709 12019199

[83] Lv QL Wang GH Chen SH Hu L Zhang X Ying G. *In vitro* and *in vivo* inhibitory effects of *glycyrrhetinic acid* in mice and human Cytochrome P450 3A4. Int J Environ Res Public Health (2015) 13:84. 10.3390/ijerph13010084 26712778 PMC4730475

[84] Lee KW Ho WS. 18β-glycyrrhetinic acid induces, UDP-glucuronosyltransferase in rats. Prot Pep Let. (2013) 20:1360–4. 10.2174/092986652012131112124033 24261979

[85] Liao S Jin X Li J Zhang T Zhang W Shi W. Effects of s*ilymarin*, g*lycyrrhizin*, and o*xymatrine* on the pharmacokinetics of *ribavirin* and its major metabolite in rats. Phytother Res. (2016) 30:618–26. 10.1002/ptr.5567 26800424

[86] Maan AA Nazir A Khan MK Ahmad T Zia R The therapeutic properties and applications of *aloe vera*: a review. J Herb Med. (2018) 12:1–10. 10.1016/j.hermed.2018.01.002

[87] Naveen P Padma J Vasudha B Gouda TS Herb-drug interaction between ethanolic extract of *aloe vera* with *glipizide* in streptozotacin induced diabetic rats. Indo Am J Pharm Res. (2016) 6:4265–9. 10.1044/1980-iajpr.151142

[88] Panahi Y Aslani J Hajihashemi A Kalkhorani M Ghanei M Sahebkar A Effect of a*loe vera* and pantoprazole on gastroesophageal reflux symptoms in mustard gas victims: a randomized controlled trial. Pharm Sci. (2006) 22:190–4. 10.15171/PS.2016.30

[89] Yang MS Yu CP Huang CY Chao PDL Lin SP Hou YC. *Aloe* activated P-glycoprotein and CYP 3A: a study on the serum kinetics of aloe and its interaction with cyclosporine in rats. J Food Func. (2017) 8:315–22. 10.1039/c6fo00938g 28009901

